# Can We Dissociate Contingency Learning from Social Learning in Word Acquisition by 24-Month-Olds?

**DOI:** 10.1371/journal.pone.0049881

**Published:** 2012-11-21

**Authors:** Colin Bannard, Michael Tomasello

**Affiliations:** 1 Department of Linguistics, University of Texas at Austin, Austin, Texas, United States of America; 2 Department of Developmental and Comparative Psychology, Max Planck Institute for Evolutionary Anthropology, Leipzig, Germany; Kyushu University, Japan

## Abstract

We compared 24-month-old children’s learning when their exposure to words came either in an interactive (coupled) context or in a nonsocial (decoupled) context. We measured the children’s learning with two different methods: one in which they were asked to point to the referent for the experimenter, and the other a preferential looking task in which they were encouraged to look to the referent. In the pointing test, children chose the correct referents for words encountered in the coupled condition but not in the decoupled condition. In the looking time test, however, they looked to the targets regardless of condition. We explore the explanations for this and propose that the different response measures are reflecting two different kinds of learning.

## Introduction

The word learning literature appears, at first glance at least, to contain contradictory findings concerning the role of social cognition in children’s word learning. It is widely accepted that words are inherently social in nature - they are shared knowledge that people use to direct one another’s attention to things in their common environment [1]. A powerful demonstration of this social dimension of word learning was provided by Baldwin and colleagues [2]. In their study, children were exposed to novel word-object pairings under two conditions. In the first “coupled” condition, an adult concurrently attending to an object with the child produced the word. In a second “decoupled” condition, the word was produced by an adult who the child could hear but not see. The child believed this hidden speaker to be on the phone. In a test phase, the child was asked comprehension questions and appeared to have learned the word in the coupled but not in the decoupled condition. It seems, then, that the children only learned the words when they were produced by an adult who was present and was jointly attending to the labeled object.

At the same time, we know from decades of study that humans are voracious contingency learners (see [3] for a summary), and that learning of sound patterns can occur even without conscious attention [4]. Associative learning is central to some accounts of word learning [5], and young children have been shown to learn word-object mappings based on contingent presentation with no social context [6], and even to perform complex inferences based on patterns of such contingencies [7]. It seems odd, then, that the absence of access to the speaker’s attentional state should completely preclude such learning.

This paper attempts to reconcile this apparent contradiction. We suggest that accounting for the Baldwin et al. result does not require one to assume a total absence of learning in the decoupled condition (in fact the authors of that paper do not make so strong a claim). We explore the possibility that children are learning associations in both conditions but that the response measure used (asking the children for explicit points) requires either some additional information or another kind of learning altogether.

The design of our study was as follows. As in [2], we exposed children to word-object pairings in a coupled and a decoupled condition, and tested whether they had learned the function of the words by asking them to point to disambiguate a referential act. We additionally tested the child’s knowledge using a preferential looking test. We predicted that as in the original study, children would fail to infer a referential function for the word in the decoupled condition. However, since there was still co-exposure, we predicted that the children would nonetheless form associations between the word (or minimally some aspect of the labeling event; we will return to this distinction in our discussion) and the object. Crucially, we expected that this associative knowledge would show up in our preferential looking test.

In preferential looking studies, the child is co-exposed to a novel word (e.g., *modi*) and an object multiple times. They are then shown a pair of objects (one of which is the just-named object) and encouraged to look for the modi. In a number of studies, young children have been shown to look preferentially to the labeled object over the distractor [8]–[9] [6], thus indicating learning of a word-object mapping. Other studies have explored conditions under which children will fail to look preferentially to a labeled object (e.g. when cues indicating that a label refers to a pictured object conflict with one another [10]). Our goal in this study was to test whether children might look preferentially even when in a more explicit test they showed no sign of having inferred this mapping.

In using preferential looking to test for awareness of a potentially non-referential relationship between a word and an object, we are building on the finding that upon hearing words people will look not only to their referents, but also to related objects [11]–[13]. For example, Moores, Laiti, and Chelazzi [14] reported that when adults were exposed to a word and to an array of objects, including an object associated with that word (e.g. they read the word *motorbike* and the array included a helmet), they looked to the associated target significantly more than to the unrelated distractors. The claim here is not that gaze behavior does not reflect children’s social learning, or their understanding of the referential intentions of others. There is in fact much evidence that gaze does reflect children’s understanding of others’ intentions from early in development (referential [15]–[16] and otherwise [17]–[18]). The point is rather that it also seems to reflect other kinds of learning. There is even evidence that gaze reflects implicit knowledge that is not available to conscious attention [19]–[20]. If we conceptualize what the child is doing during a preferential looking study as a search for the referent, we might reasonably expect that search to reflect information that is not exclusively semantic. Sabbagh and Shafman found [21] that children formed episodic memories linking words to objects, even when that knowledge was not reflected in the answers the children gave to specifically semantic questions about the words. We might expect such knowledge to affect a child’s visual search for a referent and thus to show up in a preferential looking test.

We therefore employed (1) a preferential looking test, presenting images with no social context at test, and (2) a disambiguation of reference test of the kind employed by Baldwin and colleagues [2]. Our prediction was that the pointing test would reveal learning of the word in the coupled condition only, but that children would nonetheless look preferentially to the target object for words encountered in both the coupled and the decoupled condition.

## Methods

### Ethics Statement

The study was approved by the Max Planck Institute for Evolutionary Anthropology Child Subjects Committee. The studies were conducted with the written informed consent of the children’s parents, and in accordance with all applicable laws and rules governing psychological research in Germany.

### Participants

Thirty-two normally developing monolingual German-speaking children of approximately 24 months of age (23–25 months, mean = 24 months; 15 boys) were included in the study. A further thirteen children had to be excluded because of parental interference (5), fussiness (4), experimenter error (1), identification of novel objects with familiar names (1), failure to produce points to either novel object (1), or directing all 8 points in a test block to the same side (1). Children were recruited from a database of parents who had agreed to their participation in studies. The sample was predominantly white and middle-class.

### Procedure

The experiment consisted of a warm-up followed by two blocks, each consisting of a training phase (one condition in the first block and one in the second block, with order counterbalanced) and a testing phase. The warm-up took place in a separate room near the testing room and lasted 10–15 minutes, during which the child played with Experimenter 2 (E2) and then Experimenter 1 (E1). After excusing herself, E2 went to the testing room, sat hidden from all but a small area of the room by a screen, and began simulating a telephone conversation. The child and their caregiver then entered the room with E1. E1 took the child across the room to a place from which E2 was visible and explained that E2 was on the phone with her grandmother. E1 then took the child and their caregiver to a table and chairs in the center of the room. The child sat on their caregiver’s lap at the table, facing the screen that hid E2, upon which two monitors were mounted at the child’s eye level. E2 observed the child via closed-circuit television.

The first training phase then began, during which E1 and the child would play with a pair of unfamiliar novel toys. E1 sat in a chair at the table at a 90° angle to the child. E1 removed the first toy from a bucket under the table and presented it to the child. The child was allowed to play with this toy for 30 seconds. E1 looked at the object while the child played with it, before eventually taking the toy from the child and handing over the next toy. If the child’s attention to the object waned, E1 would handle the object in order to bring their attention back to it. E2 continued to simulate a telephone conversation for this phase, but limited vocalizations to “hmm!” or “aha!”. While the child played with one of the two toys, it was labeled with a novel non-word under one of the following conditions.

Coupled: In this condition, E1 looked at the toy and produced its label (e.g., “ein Modi!”) while the child attended to the object. This was produced a total of three times at approximately 10-second intervals over the 30 seconds.

Decoupled: In this condition E2 (who was monitoring the child via closed-circuit television) said the name of the toy (e.g., “ein Modi!”) while the child attended to it. This was produced a total of three times at approximately 10-second intervals over the 30 seconds.

Each child was trained in both conditions. Whether the first or the second toy in each block was labeled was counterbalanced across conditions. The two labels used – “modi” and “dena” – occurred equally in each condition and ordering. The order of the four toys was kept constant, meaning they occurred equally with each label, each training/testing ordering, and each condition. For one participant a fourth label was produced in error at the end of the 30-second period in the decoupled condition.

Two kinds of test were administered for each block, with their order counterbalanced over the orderings of training conditions. The experimenter conducting the test was counterbalanced across conditions, with half of the children tested by E1 (the experimenter who had been present when the novel toy was played with) and half by E2 (the experimenter who had been hidden when the novel toy was played with). The non-testing experimenter concealed herself behind the screen for the duration. Both tests began with the child being told to attend to the screens in front of them and proceeded as follows.

Pointing: The testing experimenter kneeled in front of the child, with her head positioned between the two monitors on the screen behind her, and asked the child to help her. She would then say, “Zeig mir doch mal bitte, wo is der/die/das NOUN” (show me please, where is the NOUN; the novel words were always used with the neuter article *das*), as the non-testing experimenter pressed a button to display images on each of the screens. If the child pointed, s/he was thanked and the images disappeared. If the child did not respond, the testing experimenter repeated “wo is der/die/das NOUN” up to 4 times more. There were 8 object pairs in total, with 4 consisting of the same two objects with which the child had just played (always the labeled object paired with the non-labeled object). The noun produced was the non-word with which that object had been “labeled.” The side where the target object appeared was alternated and these trials were interspersed in a set order with 4 pairs of familiar objects. The child’s response was recorded from an overhead camera.

For two children it was necessary to cut one of the pointing test blocks short due to fussiness. One child who failed to respond to the first request for a point on at least one trial for either object was replaced, so all children analyzed produced at least a single codeable point. Children produced an average of 3.6 points in the coupled condition, and 3.7 points in the decoupled condition. A paired *t* test confirmed that there was no difference between conditions in the number of points provided (*t*(31) = 0.533, p = 0.60, *d* = 0.059). The number of prompts used to elicit the point was 1.14 in the coupled condition and 1.13 in the decoupled condition. A paired *t* test confirmed that there was no difference between conditions in the number of prompts provided (*t*(30) = 0.198, p = 0.84, *d* = 0.054).

Preferential looking: The testing experimenter asked the child to watch the screens while she read a book and then sat behind the child and parent where she was not visible to either. When the non-testing experimenter (who was monitoring the child’s attention) pressed a button, an image of an object appeared on each screen and remained there for 6 seconds. After 2 seconds a recorded voice (the testing experimenter, which could be E1– the present-during-training experimenter – or E2– the hidden-during-training experimenter) said “wo ist das NOUN?” (where is the NOUN). In each case the noun referred to one of the displayed objects; the other object was a distractor. After each object pair the non-testing experimenter would cause a face between the two screens to light up in order to draw the child’s gaze back to the center. There were 12 object pair displays in total, with 4 consisting of the two objects with which the child had just played (the labeled object paired with the non-labeled object; these trials were interspersed in a set order with 8 pairs of familiar objects), and the spoken noun being the non-word with which that object had been “labeled.” The recordings for the two novel labels for a given experimenter were created by splicing the noun into the same recording and timed so that the onset of the target noun occurred at the same time point (2.67 seconds following the first appearance of the objects for one speaker and 2.70 seconds for the other. Each recording and each speaker occurred equally in each condition). The side on which the target object appeared was varied. The child’s gaze was recorded using a camera placed equidistant between the two monitors.

### Coding

A research assistant who was blind to the location of the target coded the recordings of the pointing test, indicating whether the child pointed left, pointed right, or produced no clear point for each image pair. Forty percent of the data was coded in the same way by a second research assistant. Agreement between coders was excellent (93.1%, κ = .881).

Another research assistant, also blind to the location of the target object, judged on a frame-by-frame basis where the child was gazing for the looking trials, indicating “looking left,” “looking right,” “looking elsewhere,” or “uncodeable.” The coder could control the speed while watching the 25 frames per second of the recordings. Twenty percent of the data was second coded. Correlation between the percentage of looks that each coder judged to be in the target direction for each participant for each time period and condition over all trials was very high (Pearson’s *r* = 0.994), indicating excellent agreement.

## Results

### Pointing

The mean proportion of trials or points made for which children pointed to the target is shown in Table 1. A paired *t* test confirmed that the difference between the number of points made to the target and those made to the distractor was significantly greater in the coupled than in the decoupled condition (*t*(31) = 2.1676, p<0.05, *d* = 0,383). Reanalyzing the data using the proportion of completed trials in which the child pointed to the target, instead of this difference score for points made, revealed essentially identical results (t(31) = 1.745, p<0.05, d = 0.365).

**Table 1:** The mean proportion of trials/points for which children pointed to the requested novel object (standard deviation in parentheses).

	COUPLED	DECOUPLED
PROP. OF TRIALS WITH POINT TO TARGET	.578 (.356)	.445 (.363)
PROP. OF POINTS MADE TO TARGET	.637 (.361)	.478 (.371)

We further analyzed our data by fitting mixed-effects logistic regression models to the data from all trials completed. Our outcome was the direction of the point, and the key predictor was the location of the target object – the extent to which the location of the target affects the location to which a child points being the natural indication of their accuracy. Child (N = 32) was included as a random effect on the intercept in all models to account for between-participant differences. In a null model with no predictors the log odds for the intercept were not significantly different from 0 (z = −0.163, p = 0.871, indicating that that the children did not have a side bias.

Our first analysis with predictors was a single model testing the interaction between condition and location of target as predictors, thus providing a test of how accuracy varied by condition. This was found to give a significantly better fit to the data than a model including only child and location of target (χ^2^(2) = 10.6, p<0.01, Log-likelihood ratio index [22]; hereafter LLRI = 0.032) and than a model with only child, location of target, and condition but no interaction (χ^2^(1) = 9.9, p<0.01, LLRI = 0.030), with the children’s accuracy being significantly less in the decoupled condition (B = −1,79, SE = 0.055, z = −3.23, p<0.01). We therefore built separate models for each condition. The addition of the location of the object to a model containing only child significantly improved the fit of the model (χ^2^(1) = 11.07, p<0.001, LLRI = 0.063) in accounting for pointing in the coupled condition, with the target object being on a particular side significantly increasing the probability that the child would point there (B = 1.276, SE = 0.377, z = 3.39, p<0.0001). The location of the object was not found to be useful in predicting where children would point in the decoupled condition (χ^2^(1) = 0.8, p = 0.371, LLRI = 0.005).

Next we looked at whether the number of prompts provided (which as noted above did not vary between conditions) might be predictive of children’s performance. We found that a model with an interaction between target location and the number of prompts did not give an improvement in fit over a model in which the only predictor was target location (χ^2^(2) = 2.56, p = 0.278, LLRI = 0.008), indicating that points produced in response to multiple points were no less likely to be correct than points produced in response to a single prompt.

Finally we looked at the impact of the various counterbalanced factors on children’s behavior. Adding an interaction between the location of the target and whether the test was performed by the experimenter who had been present during training or the experimenter who had been hidden did not give an improvement in fit over a model with only location of target included. This applied for either the full data set (χ^2^(2) = 0.13, p = 0.938, LLRI = 0.0004) or the coupled (χ^2^(2) = 0.62, p = 0.735, LLRI = 0.0037) or decoupled (χ^2^(2) = 1.87, p = 0.3926, LLRI = 0.0111) condition data in isolation. We also looked at whether the order in which the tests occurred (pointing and then looking or looking and then pointing) affected accuracy. Adding an interaction between the location of target and the order of test gave a marginal improvement in fit for the coupled data (χ^2^(2) = 5.07, p = 0.079, LLRI = 0.031) with participant accuracy being slightly higher when pointing came first, but not significantly (B = 1.172, SE = 0.772, z = 1.517, p = 0.129), but gave no improvement for the decoupled data (χ^2^(2) = 3.676, p = 0.159, LLRI = 0.0218). The absence of word knowledge in the decoupled condition thus cannot be attributed to prior exposure to the looking test.

### Looking

**Figure 1:**
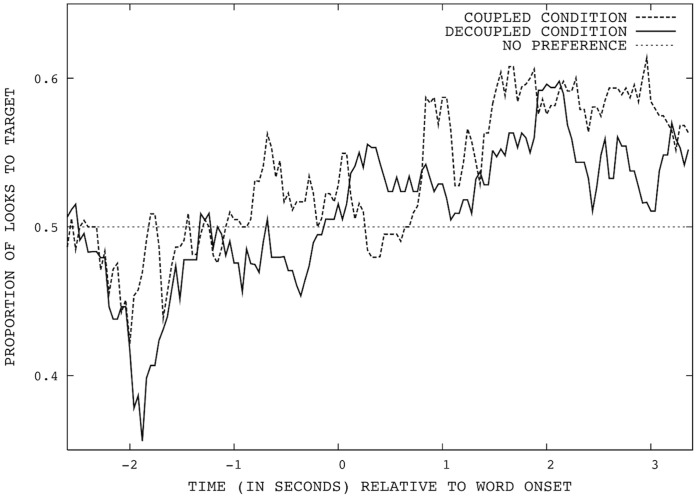
Mean proportion of children’s looks directed at the target object (word onset is at 0).

The average proportions of looks that were toward the target over the 6 seconds is shown in Figure 1. To test for changes in looking preference resulting from hearing an object label, we compared the child’s behavior in the 2600 milliseconds immediately prior to word onset (during which the object pairs were displayed on the screen), with their behavior in the 2600 ms after. We calculated the proportion of the 25 frames per second in these time windows in which the child’s looks towards either object were made to the target. We then compared the children’s looking to the target before the word onset with their looking after. Comparing before and after in this way controls for any gaze bias that is independent of the production of the label (such as a preference for attending to objects that have previously been labeled [23]). We will return to this point in our discussion. In the coupled condition, the children made an average.491 (SD = .147) of these looks to the target before word onset, compared to.554 (SD = .198) in the 2600 ms after word onset. In the decoupled condition, the children made an average.461 (SD = .158) of their looks to the target before word onset, compared to.542 (SD = .213) after word onset. We conducted a 2×2 analysis of variance for these proportions with time and condition both as within-subject factors. The analysis found a main effect of time (before word onset or after; F(1,31) = 6.019, p = 0.02, *r* = .163) but no effect of condition (F(1,31) = .269, p = 0.607, *r* = .009) and no interaction between time and condition (F(1,31) = 0.095, p = .760, *r* = .003). This indicates that the children showed a significantly greater preference for looking to the target after word onset, regardless of condition. We performed one-sample *t* tests to ensure that the difference found was not a result of a pre-word-onset preference for the distractor. These confirmed that looking to the target was significantly above chance (50%) after word onset (*t*(31) = 1.81, p = 0.04, *d* = .319), but no different from chance before. To allow time for the programming of eye saccades, some previous researchers have excluded as much as the first 300 ms [24] post-word onset from their analyses. We thus conducted an additional analysis in which the first 300 ms were excluded from both 2600 ms windows. The same pattern of results was found. Finally, the same pattern of results was found when we reduced the post-word window by 800 ms to 1800 ms, or extended it by 800 ms to include the full period for which the objects remained on-screen.

As a further check that the children’s looking toward the target objects was indeed a result of the label being produced, we performed a word-contingent switching analysis (see, for example, [25]). In such an analysis, a child’s looking behavior is classified as either correct or incorrect. If they are looking at the distractor at word onset and they switch within a specified word window (but not within the first 300 ms), or if they are looking at the target and stay looking at the target throughout the window, then their response is classified as correct. If they switch from the target within the window, or stay looking at the distractor for full duration, then the response is classified as incorrect. We employed the same window (1800 ms) as Fernald and colleagues [25]. We calculated the proportion of trials on which participants were correct, and then tested whether the participants were correct at a level greater than chance. They were correct an average of 59.9% of the time in both the coupled condition (*t*(30) = 1.436, p = 0.081, *d* = 0.2578396) and in the decoupled condition (*t*(31) = 1.917, p = 0.032, *d* = 0.3387843). A *t* test confirms that there was no significant difference between the accuracy rates in the two conditions (*t*(30) = 0.1584, p = 0.875, *d* = 0.04).

### The Relationship between Pointing and Looking

The two sets of analyses above suggest a dissociation between the knowledge displayed in the different tests. To further explore this, we tested whether children that showed evidence of a word-object mapping in the looking test were more likely than other children to point correctly. We did this by subtracting each child’s looking preference before word onset (in our looking experiment) from their looking preference after word onset to give a single difference score and adding this to the logistic regression models used to analyze the pointing data – including an interaction between the location of the target object (our measure of accuracy) and the difference score. Including this interaction offered a significant improvement in fit over a model including just child and target location (χ^2^(2) = 16.399, *p<*0.001, LLRI = 0.049). However, yet a further improvement in fit was given by adding a three-way interaction between target location, difference score and condition (χ^2^(4) = 13.045, *p<*0.05, LLRI = 0.041), indicating that the extent to which looking behavior was predictive of pointing accuracy varied with condition. We therefore built separate models for the two conditions. For the coupled data, adding an interaction between target location and the difference score gave an improvement in fit over a model with just child and target location included (χ^2^(2) = 14.189, *p<*0.001, LLRI = 0.086), with the probability that a child would point accurately increasing as the child’s difference score increased (B = 6.65, SE = 1.88, z = 3.546, p<0.001). For the decoupled data, however, the addition of such an interaction gave no improvement in fit (χ^2^(2) = 3.47, *p* = 0.176, LLRI = 0.021). This indicates that looking behavior is useful in predicting pointing behavior for word-object pairings encountered under coupled conditions but not for those encountered under decoupled conditions.

## Discussion

There is uncertainty in the cognitive sciences as to what processes support word learning. We set out to explore the role of word-object association. We found (replicating [2]) that children do not show explicit knowledge of words when merely co-exposed to words and objects. Crucially, however, we found in a preferential looking test that the same children showed a significant preference for looking toward the target object when they heard the label, regardless of whether they had encountered a referential use of that label or had merely been co-exposed to the word/object pair. Our analysis showed that children preferentially looked to the target object only after label onset, indicating that it was not simply a preference for attending to a previously-labeled object over the unlabeled object with which each target was paired.

We interpret our results as confirmation that word-object association has a role in word learning – we found that children’s looking behavior was predictive of their pointing behavior when learning occurred in the coupled (but not the decoupled) condition. However, we also interpret the result as evidence that such association is only one aspect of what is required for a child to show explicit knowledge of a word’s meaning – while it appears to be a necessary condition of knowing a word, it is not sufficient to support at least one behavioral response. This, to our knowledge, is the first work to find such a dissociation for children’s word learning. It joins a growing body of research indicating that different behavioral responses reveal different knowledge, both linguistic and non-linguistic and in both children [26]–[28] and adults [29]–[31].

The first theoretical question raised by our study concerns what the child is missing experientially in the decoupled condition. The essential difference is that in the coupled condition the adult is attending to the object and to the child while producing the label. A great deal of other work supports the observation that speaker’s gaze is important in children’s learning of words [32]–[34]. A common way of understanding the importance of gaze is as marking for the child that the contingency between the production of the label and the presence of (or the child’s attending to) the object is deliberate. It is possible that this lack of intentionality results in the child’s failure to learn a symbolic representation in our study. Perhaps the leanest way to think about this is that the child encounters a great many contingencies between sounds and objects, many of which may be misleading rather than indicative of a pattern that can be generalized to other situations. That the contingency is intended (or caused) by an adult speaker reduces the probability that it was purely coincidental and encourages the child to encode it as a word meaning.

Another possibility is that it is the sharedness of the label that is important – that the children understand something about the conventional nature of words. Nelson makes a useful distinction when she writes [35] that there are “three different kinds of meaning: subjective meaning, established within the individual’s meaning system as a whole; shared meaning, established between two or more speakers within a given context; and objective meaning, a repository of the culture” (pp.11–12). Word-object mapping is purely subjective – it is an individual’s awareness of a relationship between a word and an object. According to this account, what is missing from the child’s knowledge when s/he has formed such a representation is an awareness of the “shared meaning” of the word – an awareness that others share the mapping (acquired by the contingency occurring within a shared attentional frame in which the child is a participant or of which the child is an observer; see, for example, [36]–[38] for examples of word learning in the latter situation). Speaker intentions play a role in such an explanation, but this account also requires that the child have some awareness of the conventional status of words. The ability of young children to track what experiences are shared and not shared has been shown in both linguistic [39] and non-linguistic contexts [40]–[41]. It has been found [42] that 24-month olds understand that object labels are shared across speakers while object preferences are not, suggesting that they understand something about the conventional status of words.

A final possibility is that children’s knowledge is specific to particular tasks or domains of action. A number of writers [27] [43] have argued that children’s knowledge should not be separated from the tasks for which it is deployed. In our study both the coupled and the decoupled condition involved social engagement, but the coupled condition involved more interaction as the present experimenter delivered the label. Similarly the pointing test involves more social engagement than the looking test. It is possible that situations of social interaction will only elicit behaviors that have been learned in similar interactive situations, and that this accounts for the pattern of responses we see. Speaking against this account, however, is the fact that the words learned in the coupled condition showed up in the looking test, where there was no social prompting. It also appears that any such an effect is not a matter of straightforward perceptual overlap between the training and testing situations as it made no difference which experimenter (previously present/previously hidden) performed the pointing test.

A second question is why the child should learn about the contingency at all in the decoupled condition. Since the label uttered is not a conventional label, it might seem better for the child to simply ignore it. We would argue that if – as we have suggested – word-object association is a component of word knowledge, it would be useful for the child to track this relationship. Although the contingent use of the label by the hidden speaker cannot be taken as conclusive evidence of a word, it does certainly increase the probability that there is a conventional relationship between the two elements, for which corroborating evidence might be provided later. Thus the stored representation of this contingency might later be combined with other evidence in order to learn a word.

A parallel discussion can be found in the literature on children’s avoidance of learning words from unreliable speakers, where it has been asked whether children simply ignore word-object pairings encountered under such conditions, or remember the event but do not integrate it into their lexical knowledge. Sabbagh and Shafman present evidence [21] that children have episodic memory for labels from unreliable speakers but do not form semantic representations from them, attributing this to an adaptive mechanism that they call “semantic gating.” If the pattern we report is the result of a related semantic “gating” in the decoupled condition, it raises very interesting questions about how the pattern seen in our study might vary with age. The ability to reject word-object contingencies as being non-conventional (or non-intentional) might be a relatively late development. Younger children might make no distinction between the words learned in our two conditions, such that both would show up in their pointing. The knowledge reflected in pointing and looking might be the same at younger ages, and diverge later.

A third important question is whether this difference is qualitative or quantitative – whether the pointing and looking-time tests reflect partially different cognitive mechanisms, or whether, due to differing demands, they simply require different strengths of representation. While we cannot rule out this second explanation, our results provide no support for it. If the representations employed differed only quantitatively, we might expect that the children who pointed accurately would also be those who looked more to the target. However we only found such a relationship in the coupled condition.

The suggestion that there are two qualitatively different mechanisms at least partially at work is consistent with the findings that intentional processes rely on different learning mechanisms [44] and brain substrates [45] [31] than do implicit ones. Three-, five-, and eight-year-olds have been found [46] to be disadvantaged relative to adults in the former kind of learning, but closer in the latter. Related evidence of a dissociation between kinds of knowledge in young children comes from findings [47] that two-year-olds are able to produce and suppress sequence knowledge that has been learned incidentally, but are not able to control information learned as explicit rules until much later [48]. As we mentioned in the introduction, implicit knowledge has also been found to affect where adults look during visual search [19]–[20]. To relate this back to the discussion above, it seems plausible that knowledge of intended contingencies or shared meanings would be under conscious control, while we might expect word-object associations to be implicit in many cases.

One question that requires further research is the effect of our decision to label only one object in each condition, meaning that our distractor objects were unlabeled. As mentioned above, our analysis showed that children’s looking to the target object following label onset was a response to the utterance in our study, and not simply a preference for attending to objects that had previously been labeled. Nonetheless, real word understanding often requires the ability to discriminate the label from other possible labels in order to uniquely determine a referent. No such discrimination was necessary in order for our children to look preferentially post-word onset in our study. It is clear that the children are showing evidence of having learned a contingency between the object and some aspect of the “labeling” event in both conditions. Hence we have discovered the dissociation we predicted. However, it is possible that the child’s association is between the object and some broader property of the utterance (some aspect of the human voice), and not necessarily the word’s distinguishing phonemes. Further work will be necessary to see whether the dissociation we have reported remains in a more demanding design where the child’s utterance contingent gaze relies on the formation of a robust representation for the sound of the word.

One final question that these results raise is methodological – given these findings, how should we evaluate children’s word learning? We would argue that we need a diverse approach. Our experiment demonstrates clearly that using an implicit measure like gaze-tracking is necessary in order to identify some kinds of knowledge in young children. On the other hand, the result also suggests that gaze-tracking should not be treated as merely a replacement for explicit tests of knowledge. Its particular value appears to be as a tool for studying different, and seemingly dissociable, aspects of linguistic knowledge.

## References

[1] Bruner J (1983) Child’s talk: Learning to use language. New York: W.W. Norton.

[2] Baldwin DA, Markman EM, Bill B, Desjardins RN, Irwin JM (1996) Infants’ reliance on a social criterion for establishing word-object relations. Child Dev 67: 3135–3153. 9071774

[3] Shanks DR (2007) Associationism and cognition: Human contingency learning at 25. Q J Exp Psychol (Hove) 60: 291–309. 17366302 10.1080/17470210601000581

[4] Seitz AR, Protopapas A, Tsushima Y, Vlahou EL, Gori S, et al (2010) Unattended exposure to components of speech sounds yields same benefits as explicit auditory training. Cognition 115: 435–443. 20346448 10.1016/j.cognition.2010.03.004 PMC2866797

[5] Smith L (2000) Learning how to learn words: An associative crane. In: Golinkoff RM and Hirsh-Pasek K, editors. Becoming a word learner: A debate on lexical acquisition. New York: Oxford University Press. 51–80.

[6] Schafer G, Plunkett K (1998) Rapid word learning by 15-month-olds under tightly controlled conditions. Child Dev 69: 309–320. 9586207

[7] Smith L, Yu C (2008) Infants rapidly learn word-referent mappings via cross-situational statistics. Cognition 106: 1558–1568. 17692305 10.1016/j.cognition.2007.06.010 PMC2271000

[8] Hollich G, Hirsh-Pasek K, Golinkoff RM (1998) Introducing the 3-D intermodal preferential looking paradigm: A new method to answer an age-old question. In: Rovee-Collier C, editor. Advances in Infancy Research, vol. 12. Norwood, NJ: Ablex Publishing Company, 355–373.

[9] Houston-Price C, Plunkett K, Harris PL (2005) Word learning “wizardry” at 1;6. J Child Lang 32: 175–189. 15779882 10.1017/s0305000904006610

[10] Houston-Price C, Plunkett K, Duffy H (2006) The use of social and salience cues in early word learning. J Exp Child Psychol 95: 27–55. 16677668 10.1016/j.jecp.2006.03.006

[11] Cooper RM (1974) The control of eye fixation by the meaning of spoken language: A new methodology for the real-time investigation of speech perception, memory, and language processing. Cognitive Psychology 6: 84–107.

[12] Huettig F, Altmann GTM (2005) Word meaning and the control of eye fixation: Semantic competitor effects and the visual world paradigm. Cognition 96: B23–B32. 15833303 10.1016/j.cognition.2004.10.003

[13] Huettig F, Altmann GTM (2011) Looking at anything that is green when hearing “frog” - How object surface colour and stored object colour knowledge influence language-mediated overt attention. Q J Exp Psychol (Hove) 64: 122–145. 20521211 10.1080/17470218.2010.481474

[14] Moores E, Laiti L, Chelazzi L (2003) Associative knowledge controls deployment of visual selective attention. Nat Neurosci 6: 182–189. 12514738 10.1038/nn996

[15] Futó J, Teglas E, Csibra G, Gergely G (2010) Communicative function demonstration induces kind-based artifact representation in preverbal infants. Cognition 117: 1–8. 20605019 10.1016/j.cognition.2010.06.003

[16] Gliga T, Csibra G (2009) One-year-old infants appreciate the referential nature of deictic gestures and words. Psychol Sci 20: 347–353. 19207689 10.1111/j.1467-9280.2009.02295.x

[17] Southgate V, Csibra G (2009) Inferring the outcome of an ongoing novel action at 13 months. Dev Psychol 45: 1794–1798. 19899933 10.1037/a0017197

[18] Sommerville JA, Woodward AL (2005) Pulling out the intentional structure of human action: The relation between action production and processing in infancy. Cognition 95: 1–30. 15629472 10.1016/j.cognition.2003.12.004 PMC3908451

[19] Pertzov Y, Zohary E, Avidan G (2009) Implicitly perceived objects attract gaze during later free viewing. J Vis 9: 1–12. 10.1167/9.6.6 19761297

[20] Walter E, Dassonville P (2005) Semantic guidance of attention within natural scenes.Vis cogn. 12: 6.

[21] Sabbagh MA, Shafman D (2009) How children block learning from ignorant speakers. Cognition 112: 415–422. 19589508 10.1016/j.cognition.2009.06.005

[22] McFadden D (1974) Conditional logit analysis of qualitative choice behavior. In: Zarembka P, editor. Frontiers in econometrics. New York: Academic Press, 105–142.

[23] Baldwin DA, Markman EM (1989) Establishing word–object relations: A first step. Child Dev 60: 381–398. 2924658 10.1111/j.1467-8624.1989.tb02723.x

[24] Lew-Williams C, Fernald A (2007) Young children learning Spanish make rapid use of grammatical gender in spoken word recognition. Psychol Sci 18: 193–198. 17444909 10.1111/j.1467-9280.2007.01871.x PMC3206966

[25] Fernald A, Swingley D, Pinto JP (2001) When half a word is enough: Infants can recognize spoken words using partial phonetic information. Child Dev 72: 1003–1015. 11480931 10.1111/1467-8624.00331

[26] Gershkoff-Stowe L, Smith LB (2004) Shape and the first hundred nouns. Child Dev 75: 1098–1114. 15260867 10.1111/j.1467-8624.2004.00728.x

[27] Samuelson LK, Schutte AR, Horst JS (2009) The dynamic nature of knowledge: Insights from a dynamic field model of children’s novel noun generalizations. Cognition 110: 322–345. 19131050 10.1016/j.cognition.2008.10.017 PMC3063609

[28] Zelazo PD, Frye D, Rapus T (1996) An age-related dissociation between knowing rules and using them. Cogn Dev 11: 37–63.

[29] Knowlton BJ, Squire LR (1993) The learning of natural categories: Parallel memory systems for item memory and category-level knowledge. Science 262: 1747–1749. 8259522 10.1126/science.8259522

[30] Knowlton BJ, Squire LR (1996) Artificial grammar learning depends on implicit acquisition of both rule-based and exemplar-based information. J Exp Psychol Learn Mem Cogn, 22, 169–181. 10.1037//0278-7393.22.1.169 8648284

[31] Zeithamova D, Maddox WT, Schnyer DM (2008) Dissociable prototype learning systems: Evidence from brain imaging and behavior. J Neurosci 28: 13194–13201. 19052210 10.1523/JNEUROSCI.2915-08.2008 PMC2605650

[32] Baldwin D (1993a) Infants’ ability to consult the speaker for clues to word reference. J Child Lang 20: 395–418. 8376476 10.1017/s0305000900008345

[33] Baldwin D (1993b) Early referential understanding: Infants’ ability to recognize acts for what they are. Dev Psychol 29: 832–843.

[34] Moore C, Angelopoulos M, Bennett P (1999) Word learning in the context of referential and salience cues. Dev Psychol 35: 60–68. 9923464 10.1037//0012-1649.35.1.60

[35] Nelson K (1985) Making sense: The acquisition of shared meaning. New York: Academic Press.

[36] Akhtar N, Jipson J, Callanan M (2001) Learning words through overhearing. Child Dev 72, 41–30. 10.1111/1467-8624.00287 11333075

[37] Akhtar N (2005) The robustness of learning through overhearing. Dev Sci 8: 199–209. 15720377 10.1111/j.1467-7687.2005.00406.x

[38] Floor P, Akhtar N (2006) Can 18-month-old infants learn words by listening in on conversations? Infancy 9: 327–339. 10.1207/s15327078in0903_4 33412677

[39] Akhtar N, Carpenter M, Tomasello M (1996) The role of discourse novelty in early word learning.Child Dev. 67: 635–645.

[40] Moll H, Tomasello M (2007) How 14- and 18-month-olds know what others have experienced. Dev Psychol 43: 309–317. 17352541 10.1037/0012-1649.43.2.309

[41] Moll H, Carpenter M, Tomasello M (2007) Fourteen-month-olds know what others experience only in joint engagement. Dev Sci 10: 826–835. 17973799 10.1111/j.1467-7687.2007.00615.x

[42] Henderson AME, Graham SA (2005) Two-year-olds’ appreciation of the shared nature of novel object labels. Journal of Cognition and Development 6: 381–402.

[43] Smith LB, Thelen E, Titzer R, McLin D (1999) Knowing in the context of acting: The task dynamics of the A-not-B error. Psychol Rev 106: 235–260. 10378013 10.1037/0033-295x.106.2.235

[44] Kemler Nelson DG (1984) The effect of intention on what concepts are acquired. Journal of Verbal Learning and Verbal Behavior 23: 734–759.

[45] Poldrack RA, Foerde K (2008) Category learning and the memory systems debate. Neurosci Biobehav Rev 32: 197–205. 17869339 10.1016/j.neubiorev.2007.07.007

[46] Minda JP, Desroches A, Church BA (2008) Learning rule-defined and non-rule-defined categories: A comparison of children and adults. J Exp Psychol Learn Mem Cogn 34: 1518–1533. 18980411 10.1037/a0013355

[47] Bremner AJ, Mareschal D, Destrebecqz A, Cleeremans A (2007) Cognitive control of sequential knowledge in 2-year-olds: evidence from an incidental sequence-learning and generation-task. Psychol Sci 18: 261–266. 17444924 10.1111/j.1467-9280.2007.01886.x

[48] Kirkham NZ, Cruess LM, Diamond A (2003) Helping children apply their knowledge to their behavior on a dimension-switching task. Dev Sci 6: 449–467.

